# Genetic parameters of sole lesion recovery in Holstein cows

**DOI:** 10.3168/jds.2022-22064

**Published:** 2023-03

**Authors:** Matthew Barden, Alkiviadis Anagnostopoulos, Bethany E. Griffiths, Bingjie Li, Cherry Bedford, Chris Watson, Androniki Psifidi, Georgios Banos, Georgios Oikonomou

**Affiliations:** 1 Department of Livestock and One Health, Institute of Infection, Veterinary and Ecological Sciences, University of Liverpool, Leahurst Campus, Liverpool, CH64 7TE, United Kingdom; 2 Scotland's Rural College (SRUC), The Roslin Institute Building, Easter Bush, Midlothian, EH25 9RG, United Kingdom; 3 Department of Clinical Science and Services, Royal Veterinary College, North Mymms, Hertfordshire, AL9 7TA, United Kingdom

**Keywords:** lameness, sole lesion, genetic parameter, wound healing

## Abstract

Sole hemorrhage and sole ulcers, referred to as sole lesions, are important causes of lameness in dairy cattle. The objective of this study was to estimate the genetic parameters of a novel trait reflecting how well cows recovered from sole lesions and the genetic correlation of this trait with overall susceptibility to sole lesions. A cohort of Holstein dairy cows was prospectively enrolled on 4 farms and assessed at 4 timepoints: before calving, immediately after calving, in early lactation, and in late lactation. At each timepoint, sole lesions were recorded at the claw level by veterinary surgeons and used to define 2 binary traits: (1) susceptibility to sole lesions—whether animals were affected with sole lesions at least once during the study or were unaffected at every assessment, and (2) sole lesion recovery—whether sole lesions healed between early and late lactation. Animals were genotyped and pedigree details extracted from the national database. Analyses were conducted with BLUPF90 software in a single-step framework; genetic parameters were estimated from animal threshold models using Gibbs sampling. The genetic correlation between both traits was approximated as the correlation between genomic estimated breeding values, adjusting for their reliabilities. A total of 2,025 animals were used to estimate the genetic parameters of sole lesion susceptibility; 44% of animals recorded a sole lesion at least once during the study period. The heritability of sole lesion susceptibility, on the liability scale, was 0.25 (95% highest density interval = 0.16–0.34). A total of 498 animals were used to estimate the genetic parameters of sole lesion recovery; 71% of animals had recovered between the early and late lactation assessments. The heritability of sole lesion recovery, on the liability scale, was 0.27 (95% highest density interval = 0.02–0.52). The approximate genetic correlation between each trait was −0.11 (95% confidence interval = −0.20 to −0.02). Our results indicate that recovery from sole lesions is heritable. If this finding is corroborated in further studies, it may be possible to use selective breeding to reduce the frequency of chronically lame cows. As sole lesion recovery appears to be weakly genetically related to sole lesion susceptibility, successful genetic improvement of sole lesion recovery would benefit from selection on this trait directly.

## INTRODUCTION

Lameness in dairy cattle is a conspicuously painful condition that is ranked as the most important animal-based indicator of animal welfare on dairy farms (Whay et al., 2003a; Bicalho and Oikonomou, 2013). Lameness is also a major barrier to productivity because it is associated with reduced milk production (Green et al., 2002; Amory et al., 2008), poorer fertility (Melendez et al., 2003; Garbarino et al., 2004), and increased risk of culling (Booth et al., 2004; Bicalho et al., 2007b). One reason lameness has such a severe impact on both welfare and productivity is the long duration of behavioral and physiological changes attributed to lameness, which can persist for weeks or even months (Whay et al., 1998; Almeida et al., 2008; Laven et al., 2008).

Lameness is highly prevalent in dairy cows in the United Kingdom (**UK**); a recent meta-analysis, using data from 27 studies published between 2000 and 2020, estimated the national prevalence to be between 30 and 40% (Afonso et al., 2020). Approximately 50% of lameness can be attributed to chronically lame cows (Archer et al., 2010; Reader et al., 2011); therefore, cases of lameness must be prevented from becoming chronic to reduce the number of lame cows.

Lameness in dairy cattle is primarily associated with foot lesions (Murray et al., 1996; Bicalho et al., 2007a; van Huyssteen et al., 2020), and 2 of the most prevalent are sole hemorrhage and sole ulcers (Murray et al., 1996; Cramer et al., 2008; Somers and O'Grady, 2015). Sole hemorrhage and sole ulcers, collectively referred to as sole lesions, are thought to represent different stages or manifestations of the same disease process (Offer et al., 2000; Lischer and Ossent, 2002). The incidence of sole lesions peaks around 3 to 4 mo after calving (Leach et al., 1997; Offer et al., 2000; Barker et al., 2009). The prevalence of mild sole hemorrhage can be exceptionally high in early lactation (Bergsten and Herlin, 1996; Capion et al., 2008; Maxwell et al., 2015), but only severe cases of sole hemorrhage are generally considered clinically significant (Leach et al., 1998), at least in the short term.

Sole ulcers are particularly time consuming and expensive to treat (Charfeddine and Pérez-Cabal, 2017; Dolecheck et al., 2018), with high rates of recurrence observed in consecutive lactations (Enevoldsen et al., 1991; Foditsch et al., 2016; Charfeddine and Pérez-Cabal, 2017). One of the primary goals of sole lesion treatment is to minimize the severity and duration of inflammation because inflammation may be associated with new bone development on the distal phalanx and an increased risk of recurrence (Lischer et al., 2002b; Newsome et al., 2016; Pedersen and Wilson, 2021).

Treatment success can be determined by visual assessment of lesion healing or the resolution of visible lameness; both are reported to have similar time frames. Approximately 60 to 70% of uncomplicated sole ulcers were covered in a layer of new horn after 4 wk (Van Amstel et al., 2003; Klawitter et al., 2019). With prompt and effective treatment, more than 75% of cows with sole lesions were no longer lame after 35 d (Thomas et al., 2015), although this was only true for 15% of cows that were chronically lame when treated (Thomas et al., 2016).

Genetic selection of dairy cattle has driven exceptional improvements in production, but there has been a genetic decline of health in dairy herds, and there is an urgent need to reverse this trend (Oltenacu and Algers, 2005; EFSA, 2009; Miglior et al., 2017). Genetic selection for lameness resistance could produce cumulative, long-term benefits to complement husbandry-based initiatives. However, just as lameness control programs include measures to ensure affected animals recover quickly and to prevent new cases (Bell et al., 2009; Leach and Whay, 2009), breeding goals should also reflect this. Ultimately, the strongest foundation from which to reduce the intractably high prevalence of lameness on dairy farms would be to select for cows with better resistance to lameness and a better ability to recover from lameness.

Historically, farmers wishing to reduce lameness in their herd through genetic improvement could only select on indirect traits such as conformation (McDaniel, 1998), but it is now recognized that it is more effective to select on direct health traits, such as foot lesion records (Egger-Danner et al., 2015). The first step toward developing selection indexes is to understand the additive genetic variance that exists in a population. The heritability of sole lesion susceptibility in dairy cattle has been estimated on the underlying liability scale to range from 0.02 to 0.09 for sole hemorrhage (Buch et al., 2011; Häggman et al., 2013; Malchiodi et al., 2017) and from 0.02 to 0.18 for sole ulcers (Huang and Shanks, 1995; Ødegård et al., 2013). These heritability estimates highlight the possibility of reducing lesion susceptibility through breeding, and foot lesion records have been directly incorporated into national selection indexes in many countries (Stoop et al., 2010; Häggman and Juga, 2013; Malchiodi et al., 2020). The heritability of sole lesion recovery, however, is unknown.

The objectives of this study were to estimate the genetic parameters relating to the recovery of sole lesions in dairy cows and to consider how this trait relates to the genetic background of sole lesion susceptibility.

## MATERIALS AND METHODS

### Study Design and Population

The study was conducted following ethical approval by the University of Liverpool Research Ethics Committee (VREC269a, VREC466ab), and procedures regulated by the Animals (Scientific Procedures) Act were conducted under a UK Home Office License (P191F589B).

A prospective cohort study was designed to record sole hemorrhage and sole ulcers at 4 timepoints during a production cycle. Data collection was conducted on 4 dairy herds (A to D) in north Wales and the northwest of England, which were selected for convenience based on the practicalities of frequent visits and assessments. Herds A to C housed lactating cows year-round, milked cows 3 times daily, and recorded 305-d milk yields of approximately 11,000 to 11,500 L. Herd D housed lactating cows year-round except for lower-yielding cows, which were grazed during the summer; cows were milked twice daily and 305-d milk yield was approximately 9,000 L. In all herds, lactating cows were housed in freestalls with deep sand beds (herds B and C), mattresses with a layer of sand (herd D), or mattresses with a layer of sawdust (herd A). All herds had rubber matting in the parlor and grooved concrete in pen passageways and the collecting yards. Parous cows in all herds were routinely foot-trimmed twice a year before drying off and 60 to 120 d after calving. In all herds, lactating cows were foot bathed after milking. Herd A foot bathed cows 3 times a week with either copper sulfate or formalin; herd B foot bathed cows twice daily with formalin, herd C foot bathed cows daily with either copper sulfate or formalin, and herd D foot bathed 3 times a week with formalin.

All animals that were registered as Holsteins and expected to calve between April and December 2019 were prospectively enrolled, with no additional inclusion or exclusion criteria applied. A total of 2,352 animals were enrolled. Data were collected by qualified veterinary surgeons during weekly or twice-weekly visits to each herd from February 2019 to July 2020. Animals were assessed at 4 timepoints: before parturition (T1-Precalving), immediately after parturition (T2-Calving), in early lactation close to peak milk yield (T3-Early), and in late lactation (T4-Late). Sample size was determined by resource constraints; all eligible animals were enrolled until the final assessments (T4-Late) began, at which point further enrollments ended as data collection at 4 timepoints simultaneously was not feasible.

### Data Collection

**Figure 1:**
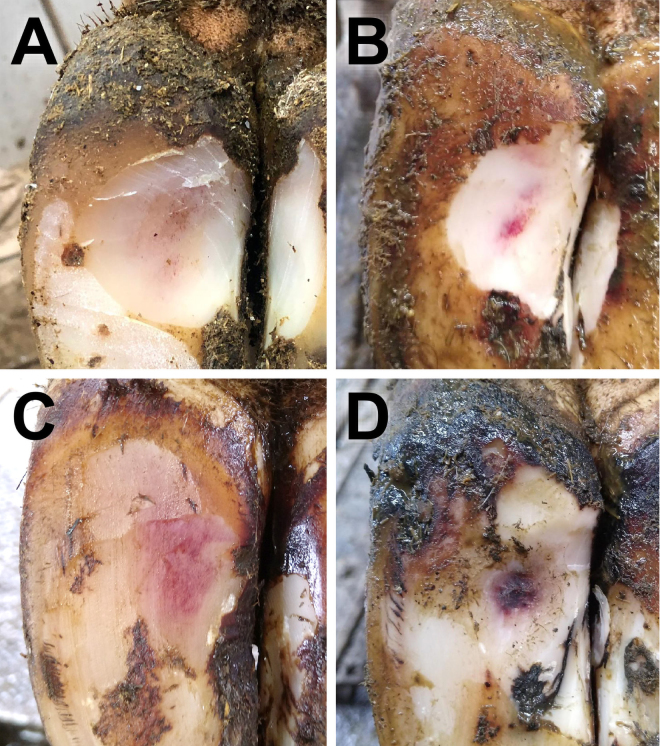
Examples of sole hemorrhage severity grading. Mild sole hemorrhage: diffuse discoloration of sole (A) or a light pink lesion <2 cm diameter (B); severe sole hemorrhage: light pink lesion ≥2 cm diameter (C) or dark pink/purple lesion of any size (D).

At each assessment, all cows were mobility scored according to a 4-point system from 0 (sound) to 3 (severely lame) (Whay et al., 2003b; AHDB, 2020). Animals were restrained in a foot-trimming crush and, if foot-trimming was not conducted during the visit, the claw horn on the sole of each foot was lightly trimmed to allow inspection of foot lesions. On each claw, sole hemorrhage and sole ulcers were recorded using case definitions as described in the International Committee for Animal Recording (ICAR) claw health atlas (Egger-Danner et al., 2020). All foot lesions were examined and recorded by qualified veterinary surgeons (over 90% by a single researcher and the remainder by 3 other researchers). Sole hemorrhage was graded as either mild: light pink lesion <2 cm diameter or diffuse discoloration of sole, or severe: light pink lesion ≥2 cm diameter or dark pink/purple lesion of any size (Figure 1). Sole ulcers were recorded as present or absent. The data collection procedure was the same at all timepoints except in the case of T2-Calving in herd C, when only hind feet were assessed to reduce the handling time of cows that had recently calved; this was only required in this herd due to the large numbers of cows calving each week.

When either severe sole hemorrhage or a sole ulcer was present, the claw was therapeutically trimmed by researchers (herds A and D), farm staff (herd C), or a combination of farm staff and professional foot-trimmers (herd B). All persons responsible for foot-trimming had completed specialist training in this area and had extensive experience. Regardless of the individual involved, a modified version of the 5-step Dutch method was used (Toussaint Raven et al., 1985), which included wider and deeper modeling of the lateral claw on hind feet than the traditional method. In all cases, therapeutic foot-trimming aimed to create a concavity in the middle of the sole around the lesion and to reduce the heel of the affected claw to redistribute load onto the unaffected claw (Mahendran and Bell, 2015). Additionally, a hoof block was applied to the unaffected claw at the discretion of the foot-trimmer; a block was applied if there was exposure of the corium, a pain response was elicited following pressure on the lesion, or the animal had impaired mobility attributable to the lesion.

### Trait Definitions

Two genetic traits were defined to reflect the overall susceptibility to sole lesion development and sole lesion recovery during lactation. The case definition for a sole lesion was “the presence of severe sole hemorrhage or a sole ulcer.”

#### Susceptibility to Sole Lesions

A binary trait (**SL-Susceptibility**) classified animals as being either susceptible or resistant to sole lesions. Records from all claws across the whole study period were combined to classify animals as “susceptible” or “resistant.” If animals were affected with a sole lesion (severe sole hemorrhage or a sole ulcer) at any assessment, they were classified as “susceptible,” regardless of the number of claws affected, the number of timepoints the lesion was present, or the total number of records for that animal. Animals were classified as “resistant” if they were unaffected with sole lesions at each assessment in a complete set of records from all 4 timepoints. Therefore, animals were unclassified by this trait if they were unaffected by a sole lesion but did not have records from all 4 timepoints. This resulted in a slight reduction in study power due to a small proportion of incomplete lesion records for animals that had otherwise always been unaffected, but provided the highest confidence in the classification of animals as “resistant.”

#### Recovery from Sole Lesions

A binary trait (**SL-Recovery**) defined animals that were affected with a sole lesion at the T3-Early assessment and that either “recovered” or remained “chronic” by T4-Late. The full data set was filtered to only include animals affected with a sole lesion at T3-Early, and animals were excluded if lesion records were missing for any claw at either T3-Early or T4-Late. Animals were classified as “recovered” if all affected claws at T3-Early were no longer affected at T4-Late, and “chronic” if at least one of the affected claws at T3-Early was still affected at T4-Late.

### Pedigrees and Genotypes

Pedigree details for the study population were extracted from the national database of dairy cattle by tracing back 7 generations for each animal. A total of 14,097 animals were included in the pedigree, including 2,109 sires with an average of 5 daughters each (range: 1 to 165). Both parents were known for 11,323 animals, one parent was known for 395 animals, and both parents were unknown for 2,379 animals. Blood samples were collected from the coccygeal vein of each animal into EDTA Vacutainers (Becton Dickinson) and used to genotype each animal with the BovineSNP50 BeadChip (Illumina Inc.). Genotypes were subsequently imputed from 50K to 80K SNP genotypes by Edinburgh Genetic Evaluation Services (EGENES) using an in-house procedure that has been developed for all national genomic evaluations of dairy cattle in the UK. Briefly, this imputation process uses the BovineSNP50 and BovineHD BeadChips (Illumina Inc.), in addition to other commercial genotyping arrays, extra gene tests, and large-effect sequence variants. Following imputation, genotypes included 79,051 SNP spanning the entire genome. Chromosomal locations of the imputed 80K SNP panel were drawn from the latest assembly of the *Bos taurus* genome (ARS-UCD 1.2; Rosen et al., 2020).

Imputed genotypes were available for 2,250 animals. Genotype quality control was implemented using the PREGSF90 program (Aguilar et al., 2014) within the BLUPF90 software suite (Misztal et al., 2018). Quality control included the removal of SNP with a call rate <0.90 (n = 10,977), SNP with a minor allele frequency <0.05 (n = 3,008), monomorphic SNP (n = 36), or SNP showing a strong deviation (>0.15) from Hardy-Weinberg equilibrium (n = 14; Wiggans et al., 2009). Additionally, animals were removed if sample call rate <0.90 (n = 63) or there were parent-progeny Mendelian conflicts (n = 20). Quality control procedures resulted in a final data set of 2,167 animals with genotypes of 65,221 SNP.

### Phenotypic Analysis

At a claw level, the differences in the proportion of claws that recovered or remained chronic were assessed with chi-squared tests to compare forelimb to hindlimb lesions, and severe sole hemorrhage to sole ulcers. At a cow level, the relationship between clinical lameness (mobility score 2 or 3) and sole lesion recovery was also assessed with chi-squared tests. The duration between T3-Early and T4-Late was compared between animals classified as recovered or chronic with a 2-tailed *t*-test. The prevalence of lameness in the study population was calculated as the proportion of lame animals (mobility score 2 or 3) at each assessment timepoint (point prevalence), and as the proportion of cows that were lame (mobility score 2 or 3) at any timepoint over the whole study period (period prevalence; Mahendran et al., 2017).

### Genetic Parameter Estimation

Before genetic analyses of SL-Susceptibility and SL-Recovery, potential fixed effects were evaluated with multivariable logistic regression of each trait in R (R Core Team, 2021). The importance of each fixed effect was determined by finding the multivariable model with the lowest Akaike information criterion. In addition to the final model parameters, continuous variables of days in milk at T3-Early and T4-Late were evaluated in the SL-Recovery model, but neither improved model fit when the duration between T3-Early and T4-Late was included as a covariate. The effect of the researcher examining and recording lesions was tested but also increased the Akaike information criterion.

Variance components were estimated for both traits (SL-Susceptibility and SL-Recovery) using threshold models to transform the binary observed phenotype to a latent liability scale (Gianola, 1982). A Markov chain Monte Carlo approach was used to obtain marginal posterior distributions for model parameters via the Gibbs sampling algorithm in the THRGIBBS1F90 program (Tsuruta and Misztal, 2006). Convergence of Gibbs sampling was assessed using the *coda* package in R (Plummer et al., 2006; R Core Team, 2021); a chain length of 500,000 samples with a 50,000 sample burn-in produced consistent results in both models. Lag correlation between consecutive samples was reduced with a thinning interval of 100; therefore, genetic parameters were estimated from the posterior distribution of 4,500 Gibbs samples.

The animal threshold model used to separately analyze both traits (SL-Recovery and SL-Susceptibility) was

[1] **λ** = **Xb** + **Z***_hys_***hys** + **Z***_a_***a** + **e**,

where **λ** is a vector of unobserved liabilities for either SL-Recovery or SL-Susceptibility; **b** is a vector of the fixed effect of parity (3 levels: first, second, and ≥ third parity) and the interval between the T3-Early and T4-Late assessments in days as a continuous covariate (included in the model for SL-Recovery only; omitted from the model for SL-Susceptibility); **hys** is a vector of the random effects of herd-year-season of calving (HYS, 12 levels); **a** is a vector of random additive genetic effects for each animal; **e** is a vector of random residual effects, and **X**, **Z***_hys_*, and **Z***_a_* are incidence matrices for **b**, **hys**, and **a**, respectively. Model convergence was improved by treating **hys** as a random effect compared to a fixed effect. Random effects were assumed to be normally distributed with a mean of zero and covariance structure of

[2] $$var\left[\begin{array}{c} hys\\ a\\ e \end{array}\right]=\;\left[\begin{array}{ccc} I\sigma_{hys}^{2} & 0 & 0\\ 0 & H\sigma_{a}^{2} & 0\\ 0 & 0 & I\sigma_{e}^{2} \end{array}\right],$$

where $\sigma_{hys}^{2}$ is the hys variance, $\sigma_{a}^{2}$ is the additive genetic variance, $\sigma_{e}^{2}$ is the residual variance, **I** is an identity matrix, and **H** is the relationship matrix incorporating pedigree and genomic information in a single-step genomic analyses framework as defined by Legarra et al. (2009), with inbreeding coefficients included in the pedigree relationship matrix (Meuwissen and Luo, 1992). The heritability on the underlying liability scale was estimated as the ratio of additive genetic variance $\left(\sigma_{a}^{2}\right)$ to the sum of the HYS variance $\left(\sigma_{hys}^{2}\right),$ additive genetic variance, and residual variance $\left(\sigma_{e}^{2}\right).$ Single-step genomic BLUP, implemented in the BLUPF90 program (Misztal et al., 2018), was used to calculate genomic estimated breeding values (**GEBV**) for each trait. The reliabilities (*REL*) of GEBV were calculated as follows (Aguilar et al., 2020):

[3] $$REL_{i}=1-\;\frac{PEV_{i}}{\left(1+F_{i}\right)\sigma_{a}^{2}},$$

where *PEV_i_* is the prediction error variance of the GEBV in animal *i* (calculated as the squared standard error of the GEBV), *F_i_* is the inbreeding coefficient of animal *i* calculated from pedigree relationships (Meuwissen and Luo, 1992), and $\sigma_{a}^{2}$ is the additive genetic variance estimated with the threshold model (Equation [1]).

To assess the genetic correlation between SL-Recovery and SL-Susceptibility, a bivariate threshold model was fitted using both traits, based on the same parameters as Equation [1]. Model convergence was unsatisfactory despite extending the chain length to 1 million samples; therefore, we estimated the approximate genetic correlation between SL-Susceptibility and SL-Recovery as the correlation between GEBV. Estimated genomic breeding values were calculated for all animals in the relationship matrix; the correlation between GEBV was calculated in the subset of animals, which had both phenotypes recorded, after adjusting for the GEBV reliabilities (Calo et al., 1973):

[4] $${\tilde{r}}_{g1,2}=r_{1,2}\;\times \;\frac{\sqrt{\left(\sum REL_{1}\right)\left(\sum REL_{2}\right)}}{\sum (REL_{1}\times \;REL_{2})},$$

where *REL*_1_ and *REL*_2_ are the reliabilities of GEBV for SL-Susceptibility and SL-Recovery, and *r*_1,2_ is the Pearson correlation between GEBV. The standard error (SE) for the approximate genetic correlation was calculated as follows:

[5] $$SE=\sqrt{\frac{1-\;{{\tilde{r}}^{2}}_{g1,2}\;}{n-2}},$$

where ${{\tilde{r}}^{2}}_{g1,2}$ is the squared approximate genetic correlation calculated in Equation [4], and *n* is the number of animals with records.

## RESULTS

### Population and Data Set Description

A total of 2,352 animals were enrolled in this study: 132 animals from herd A, 432 animals from herd B, 1,549 animals from herd C, and 239 animals from herd D. Details of the timing of each assessment timepoint and the number of animals with foot lesion records at each timepoint are provided in Table 1. Table 2 displays the point prevalence of lameness from mobility scoring (lameness defined as mobility score 2 or 3) at each timepoint, in addition to the period prevalence from the whole study period (Mahendran et al., 2017). Denominators for lameness prevalence differ slightly from the number of cows with foot lesion records (Table 1), due to instances where either mobility scoring or foot lesion inspection was not possible at an assessment. The highest frequency of sole lesions was at T3-Early; details are provided in Table 3.

**Table 1:** Details of data collection at each assessment timepoint by herd, including the timing of each assessment relative to parturition and the number of cows from which foot lesions were recorded

Item	T1 (Precalving)	T2 (Calving)	T3 (Early)	T4 (Late)
Mean (SD) timing of assessment relative to parturition (d)	−56.5 (22.3)	+5.4 (2.9)	+84.0 (13.9)	+199.5 (30.5)
Herd				
A	128	124	124	116
B	432	401	398	375
C	1,544	1,447	1,398	1,243
D	237	214	212	203
Total	2,341	2,186	2,132	1,937

**Table 2:** The prevalence of lameness from mobility scoring (lame cows defined as mobility score 2 or 3) at each timepoint (point prevalence) and for the whole study period (period prevalence)

Herd	Point prevalence				Period prevalence
	T1-Precalving	T2-Calving	T3-Early	T4-Late	
A	4.7%	11.4%	17.7%	14.2%	33.3%
	(6/128)	(14/123)	(22/124)	(16/113)	(44/132)
B	5.2%	8.4%	4.9%	5.9%	18.8%
	(22/423)	(33/391)	(19/388)	(22/373)	(81/432)
C	8.6%	9.6%	7.5%	10.7%	22.9%
	(126/1,468)	(139/1,453)	(103/1,374)	(132/1,238)	(354/1549)
D	11.5%	8.8%	11.2%	12.6%	23.4%
	(27/234)	(18/204)	(23/206)	(25/198)	(56/239)

**Table 3:** Prevalence (frequency) of sole lesions in each animal at each assessment timepoint (using the most severe sole lesion from all claws)^1^

Sole lesion severity	Assessment timepoint			
	T1-Precalving	T2-Calving	T3-Early	T4-Late
No sole hemorrhage or sole ulcer	65.7% (1,538)	66.0% (1,443)	40.7% (868)	44.9% (870)
Mild sole hemorrhage	24.3% (568)	25.9% (566)	31.7% (676)	34.9% (676)
Severe sole hemorrhage	6.3% (148)	5.6% (122)	21.4% (457)	14.3% (277)
Sole ulcer	3.7% (87)	2.5% (55)	6.1% (131)	5.9% (114)
Total	2,341	2,186	2,132	1,937

1 In all analyses, animals with no sole hemorrhage or sole ulcer, or mild sole hemorrhage were considered unaffected; animals with severe sole hemorrhage or a sole ulcer were considered affected.

The numbers of animals in the final study populations for the genetic analysis of each trait are provided in Table 4. A total of 2,025 animals were used to estimate the genetic parameters of SL-Susceptibility. Not all animals were classified for this trait because animals were excluded if they were unaffected with a sole lesion but did not have complete records from all 4 timepoints. Genetic parameters of SL-Recovery used records from 498 animals. For analysis of this trait, the full data set (n = 2,352) was first filtered to include only animals affected with a sole lesion at T3-Early, and that had been assessed again at T4-Late (n = 528). Finally, only animals that had lesion records from all 8 claws at both T3-Early and T4-Late were included (n = 498). The final cohort of 498 animals corresponded to 694 affected claws.

**Table 4:** Summary of the 2 genetic traits with details regarding trait classification and frequency of animals assigned to each class

Trait	Phenotype	Definition	Frequency
Sole lesion susceptibility (SL-Susceptibility)	Resistant (= 0)	No severe sole hemorrhage or a sole ulcer on any claw, at all assessments, with no missing records	1,136
	Susceptible (= 1)	Severe sole hemorrhage or a sole ulcer on at least one claw, at any number of assessments	889
Sole lesion recovery (SL-Recovery)	Chronic (= 0)	Severe sole hemorrhage or a sole ulcer at the early lactation assessment (T3-Early) and severe sole hemorrhage or a sole ulcer still present on the same claw at the late lactation assessment (T4-Late)	146
	Recovered (= 1)	Severe sole hemorrhage or a sole ulcer at the early lactation assessment (T3-Early) and no severe hemorrhage or a sole ulcer on the same claw at the late lactation assessment (T4-Late)	352

### Phenotypic Analysis

**Figure 2:**
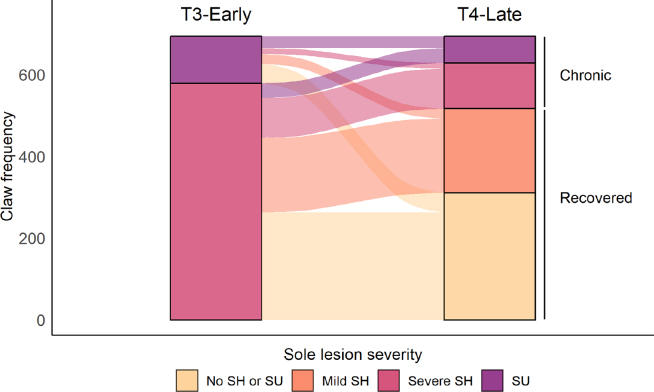
Progression of sole lesion severity between the early lactation (T3-Early) and late lactation (T4-Late) assessments was used to define the sole lesion recovery trait (SL-Recovery). Data are presented at the claw level for a total of 694 claws on 498 animals. SH = sole hemorrhage; SU = sole ulcer.

Of the claws affected with a sole lesion at T3-Early, 74.4% (517/694) had recovered by T4-Late; the outcome of each sole lesion is displayed in Figure 2. Forelimb claws were more likely to recover than hindlimb claws [87.3% (96/110) vs 72.1% (421/584), χ^2^ = 11.232, df = 1, *P* < 0.001]; recovery of severe sole hemorrhage occurred more frequently than recovery of sole ulcers [76.9% (445/579) vs 62.6% (72/115), χ^2^ = 10.251, df = 1, *P* = 0.001]. The SL-Recovery trait was defined at the animal level, and animals were only considered to have recovered if all affected claws at T3-Early were no longer affected at T4-Late. Of the 352 animals in which all affected claws had recovered (SL-Recovery = “recovered”), 262 animals had only been affected on 1 claw, 79 animals on 2 claws, 9 animals on 3 claws, and 2 animals on 4 claws. Of the 146 animals that were considered to have been chronically affected (SL-Recovery = “chronic”), sole lesions were still present at T4-Late on all affected claws in 93 animals; 53 animals had claws that recovered and claws that remained chronic, and these animals were all classified as “chronic” for the SL-Recovery trait.

In the cohort of animals used to analyze SL-Recovery, 11.5% (56/485) of animals were lame according to mobility scoring (mobility score 2 or 3) at T3-Early and 12.0% (58/482) at T4-Late. A lower proportion of cows that were classified as “recovered” had been lame at T3-Early compared with cows classified as “chronic” [9.4% (32/340) vs. 16.6% (24/145), χ^2^ = 5.074, df = 1, *P* = 0.024]. Similarly, a lower proportion of cows that were classified as “recovered” were lame at T4-Late compared with cows classified as “chronic” [7.6% (26/342) vs. 22.9% (32/140), χ^2^ = 21.838, df = 1, *P* < 0.001]. The mean interval between the T3-Early assessment and T4-Late was 115.2 d (SD: 33.9). On average, this interval was longer in “recovered” animals (mean: 118.0 days, SD: 35.6) compared with “chronic” animals [mean: 108.5 d, SD: 28.5; *t*(496) = 2.859, *P* = 0.004]. The interval between T3-Early and T4-Late was correlated with DIM of T3-Early (Pearson correlation coefficient −0.47; 95% CI −0.54 to −0.40).

### Genetic Parameters

Heritability was calculated during each round of Gibbs sampling. For SL-Susceptibility, the posterior distribution had a mean of 0.25 [95% highest density interval (**HDI**): 0.16 to 0.34]; for SL-Recovery, the posterior distribution had a mean of 0.27 (95% HDI: 0.02 to 0.52). Details of the variance component estimates are provided in Table 5. The bivariate model did not converge despite an extended chain length of 950,000 rounds (after a 50,000 burn-in); therefore, the genetic correlation between SL-Susceptibility and SL-Recovery could not be estimated directly. The genetic correlation between SL-Susceptibility and SL-Recovery was approximated from the correlation between GEBV for the 498 animals with both phenotypes recorded, after adjusting for the GEBV reliabilities. The GEBV reliabilities were low for both traits with an average reliability of 0.32 for SL-Susceptibility and 0.21 for SL-Recovery. The approximate genetic correlation between SL-Susceptibility and SL-Recovery was −0.11 (95% CI: −0.20 to −0.02).

**Table 5:** Additive genetic variance $\left(\sigma_{a}^{2}\right),$ herd-year-season variance $\left(\sigma_{hys}^{2}\right),$ residual variance $\left(\sigma_{e}^{2}\right),$ and narrow-sense heritability (*h*^2^) estimates for 2 traits: overall susceptibility to sole lesions (SL-Susceptibility) and recovery from sole lesions (SL-Recovery)^1^

Trait	$\sigma_{a}^{2}$	$\sigma_{hys}^{2}$	$\sigma_{e}^{2}$	*h*^2^
SL-Susceptibility	0.42	0.25	1.04	0.25
	(0.25–0.63)	(0.04–0.60)	(0.91–1.09)	(0.16–0.34)
SL-Recovery	0.48	0.11	1.02	0.27
	(0.02–1.20)	(0.0001–0.34)	(0.85–1.20)	(0.02–0.52)

1 Estimates refer to the posterior mean (95% highest density interval) from Gibbs sampling.

## DISCUSSION

### Key Results and Interpretation

We used a data set of accurately collected foot lesion records to define a novel trait relating to the recovery of sole lesions in Holstein cows (SL-Recovery). We estimate SL-Recovery to have a heritability of 0.27 on the liability scale; therefore, the potential exists to breed cows that can more effectively recover from sole lesions.

Reducing the prevalence of lameness is a key priority for the UK dairy industry (GB Cattle Health and Welfare Group, 2020; Rioja-Lang et al., 2020). It is suggested that producers aim for more than 75% of lame cows to recover between consecutive (e.g., monthly) mobility scores (Green, 2012), but as approximately 50% of lameness prevalence can be attributed to chronically lame cows, it is likely that many farms do not currently achieve this (Archer et al., 2010; Reader et al., 2011). Although early identification and treatment of lame cows are correctly regarded as the most important interventions required to meet this target (Bell et al., 2009; Leach et al., 2012; Groenevelt et al., 2014), breeding cows that can recover more quickly and effectively from sole lesions would also be advantageous. Additionally, longevity is currently a key priority of many breeding strategies because it is closely related to the environmental impact and profitability of dairy farms (Boulton et al., 2017; Grandl et al., 2019). As lameness has been associated with a greater risk of culling (Sprecher et al., 1997; Booth et al., 2004; Bicalho et al., 2007b), genetic selection for effective sole lesion recovery would also benefit those aiming to breed cows for a longer productive life.

The healing of claw horn lesions in cattle is similar to the secondary intention healing of cutaneous wounds (Azarabad et al., 2006; Shearer et al., 2015). The rate of ear punch hole closure has been used to investigate the genetics of cutaneous wound healing in mice, which has been characterized as a complex trait (Masinde et al., 2001) with an estimated heritability of 0.29 (Nicod et al., 2016). Wound healing is a “dynamic, interactive process involving soluble mediators, blood cells, extracellular matrix, and parenchymal cells” (Singer and Clark, 1999); therefore, there are abundant opportunities for genetic influence. Notably, this genetic influence has been a therapeutic target since the late 1990s, when gene therapy was considered a promising approach to promoting wound healing (Eming et al., 1997). However, single-gene targets (such as growth factors) showed only modest responses, which was attributed to the complexity of the healing process (Eming et al., 2014). As such, research in this field is now focused on the identification of the full spectrum of wound-healing “driver genes” to advance this area (Tang et al., 2021); ultimately, this may lead to a clearer understanding of the underlying gene pathways involved.

One possible explanation for the delayed recovery of sole ulcers is the development of complicating secondary infections with bacteria such as treponemes, which are more frequently associated with bovine digital dermatitis (Evans et al., 2011; Sykora et al., 2015). This complication could conceivably have a genetic background because genotype has been associated with the microbiome of chronic wounds in humans (Tipton et al., 2020) and foot skin microbiome in cattle (Bay et al., 2021). Another possible mechanism for genetic influence on sole lesion recovery is via IGF-1, which is a major promoter of wound healing (Garoufalia et al., 2021). In cattle, serum IGF-1 concentration has been demonstrated to be highly heritable (Davis and Simmen, 1997) and specific mutations have been identified in the *IGF1* gene (Mullen et al., 2011). However, as IGF-1 concentration also correlates with negative energy balance and body condition, recovery of sole lesions could be affected by the timing of lesion development during lactation (Fenwick et al., 2008; Akbar et al., 2015). Future studies relating to sole lesion recovery in dairy cattle would therefore benefit from minimizing the variation around the lactation stage at which healing is assessed.

We were unable to directly estimate the genetic correlation between SL-Susceptibility and SL-Recovery because the genetic variance of SL-Recovery could not be estimated with a bivariate model. Consequently, we estimated the approximate genetic correlation between SL-Susceptibility and SL-Recovery by calculating the correlation between the GEBV for each trait in animals that had both phenotypes recorded, adjusting for the GEBV reliabilities. Correlation between breeding values is only equivalent to genetic correlation when the accuracy of the GEBV is 100% (Koenig et al., 2005). Given the low reliabilities of GEBV, which were expected due to the small study population, we are cautious in our interpretation of this result. The approximate genetic correlation between SL-Susceptibility and SL-Recovery had a 95% CI of −0.20 to −0.02. We interpret this result to suggest that the genetic correlation between SL-Susceptibility and SL-Recovery is negative but very weak; therefore, these traits appear to have relatively distinct genetic backgrounds. This result was unexpected and we anticipated that these traits would be strongly genetically correlated because common biological pathways could plausibly underlie both traits. For example, genes related to keratinization and inflammation pathways have previously been linked with sole lesion susceptibility (Swalve et al., 2014; Sánchez-Molano et al., 2019; Lai et al., 2021), and these could plausibly also be involved in sole ulcer healing (Hendry et al., 2001). Nevertheless, our results do not suggest that sole lesion recovery is strongly genetically correlated with sole lesion susceptibility, although this result should be interpreted cautiously pending replication in further studies.

A practical implication of the apparently weak genetic correlation between SL-Susceptibility and SL-Recovery is that genetic selection for reduced susceptibility to sole lesions would be expected to only have a minimal effect on improving sole lesion recovery (Shook, 1989). Therefore, to effectively breed cows that can recover more quickly from sole lesions, this phenotype would need to be specifically recorded so that it could be utilized in national genetic evaluations. It is advised that cases of sole ulcers be re-examined within 30 d following treatment (Van Amstel et al., 2003), so one approach to obtain a phenotype of sole lesion recovery would be to record details of all follow-up assessments in farm records. Although this would be theoretically achievable, it is admittedly optimistic and, in general, lameness is poorly recorded on farms compared with other health conditions (Zwald et al., 2004; Leach et al., 2010; Parker Gaddis et al., 2014). A more realistic solution may be to use lesion records from professional foot-trimmers, as is the case for national genetic evaluations in other countries (Stoop et al., 2010; Häggman and Juga, 2013; Malchiodi et al., 2020). The inclusion of foot lesion records in genetic evaluations would be expected to improve existing genetic selection indexes for reduced lameness (Koenig et al., 2005; van der Linde et al., 2010; Ødegård et al., 2015), and, assuming a reasonable accuracy and consistency in recording, there may be scope to define a trait similar to SL-Recovery based on repeated foot lesion records for each animal. However, as national genetic evaluations use linear models, and therefore traits are defined on the observed rather than the underlying liability scale, further analysis of SL-Recovery within this framework would be required.

The heritability of SL-Recovery had a large uncertainty estimate (95% HDI: 0.02 to 0.52), so our results are also compatible with a very small or substantially larger true heritability, which would affect the expected response to selection and success of breeding programs. The large uncertainty around the heritability estimate is due, in part, to the small study population used to analyze this trait (n = 498), but there are other sources of noise relating to this phenotype that could contribute to this uncertainty. We discuss these further in the following section.

### Study Strengths and Limitations

One of the strengths of this study was the accuracy and detail of foot lesion recording, which allowed the outcome of lesions to be determined from sequential assessments; however, there were some limitations to our study design that may have affected the accuracy of this classification.

Studies that have assessed the healing rate of sole lesions following different treatment protocols monitored lesion outcomes at multiple timepoints (Lischer et al., 2002a; Thomas et al., 2015; Klawitter et al., 2019). We recognize that this would be a more robust approach to judge the recovery of sole lesions than a single follow-up assessment, but our priority was to use our available resources to maximize the number of enrolled animals and allow the estimation of genetic parameters. For example, the largest sample size of animals with sole lesions in the previously referenced studies with multiple follow-up assessments was 83 animals (Thomas et al., 2015); this would have been insufficient for our objectives. Therefore, we accept that a single follow-up assessment of a sole lesion has limitations regarding the ability to definitively determine healing progress.

We were not recording the spontaneous healing of sole lesions because all animals with sole lesions were therapeutically trimmed and the unaffected claw was blocked when considered necessary. This treatment protocol reflects common practice on UK dairy farms (Horseman et al., 2013), but it does not represent the best approach to the treatment of sole lesions, which includes administering nonsteroidal anti-inflammatory drugs (Thomas et al., 2015; Pedersen and Wilson, 2021). One source of extraneous noise in our data may have been inconsistencies between foot-trimmers in terms of whether unaffected claws were blocked, as this is likely to affect the recovery rate (Thomas et al., 2015). As the person responsible for foot-trimming depended on the herd, we could not control for this in our analysis beyond the inclusion of herd in the statistical model.

Wound healing is a continuous process and the point at which a sole lesion could be regarded as having recovered is not absolute. A consensus is that, in the absence of complicating factors, mild-to-moderate sole ulcers should be covered by a thin layer of new horn after 30 d, and severe lesions after 40 to 60 d (Shearer and van Amstel, 2017). The mean interval between the T3-Early assessment and T4-Late was 115.2 d (SD = 33.9) and the first percentile was 70 d; therefore, we consider the interval to have been sufficiently long for uncomplicated sole lesions to appear visibly healed. However, the interquartile range of the duration between T3-Early and T4-Late was 90 to 126 d, so in half of our study population, there was a ≥5-week difference in the interval between lesion identification and outcome assessment. We observed a significant univariable association between this interval and whether animals were considered to have recovered. We included the interval between T3-Early and T4-Late as a covariate in the animal threshold model used to estimate the heritability of SL-Recovery, which we believe will have mitigated the influence on the heritability estimate, at least to some extent. There would be benefit in future studies attempting to standardize the duration between lesion diagnosis and recovery assessment, and this may allow a more granular phenotype, such as degree of recovery, to be evaluated. The association of duration between T3-Early and T4-Late with SL-Recovery could also reflect how we classified the outcome of sole lesions, which meant that a sole ulcer was considered to have not fully recovered if severe sole hemorrhage was observed at T4-Late, despite the sole ulcer having epithelialized. We grouped severe sole hemorrhage and sole ulcers to create a trait with sufficient numbers in each class to reasonably estimate the genetic parameters of sole lesion recovery, but future studies of a sufficient sample size to assess sole ulcers independently of sole hemorrhage would be worthwhile.

In addition to the variation in the interval between T3-Early and T4-Late, there was a dispersion of both assessment timepoints relative to parturition. We consider the timing of T3-Early to be of particular clinical relevance because it relates to when sole lesions developed during lactation and the timing of therapeutic intervention. It has been shown that cows that develop sole lesions in early lactation heal more quickly and respond better to corrective trimming and foot blocking, with this response declining over time (Thomas et al., 2015). It is also probable that lesions that were identified later in lactation were more likely to represent chronic lesions, and the recovery of chronic lesions is poorer than that of acute cases (Leach et al., 2012; Groenevelt et al., 2014; Thomas et al., 2016). Furthermore, the moderate negative correlation between the timing of T3-Early and the interval between T3-Early and T4-Late meant that sole lesions recorded later in lactation also tended to have a shorter duration until the outcome was assessed, further complicating the interpretation of our results.

We acknowledge that the interpretation of sole lesion recovery in our data is complicated by several factors, and this is an important context in which to consider our results. However, if the recovery of sole lesions is ever going to be deducible from farm records such that it could be incorporated into national genetic evaluations, the ability to recognize a heritable trait from an admittedly noisy data set is encouraging.

### Generalizability

This study included only 4 dairy herds which, despite all being commercially managed with operating practices common to many British dairy farms, cannot be considered representative of the full spectrum of dairy farms. Within these 4 herds, 3 were operating relatively intensive systems of zero-grazing and 3-times-a-day milking. We did not observe any differences in trends between these 3 farms and the remaining herd, which was managed with a combination of housed and grazed groups and had lower milk production.

The overall period prevalence of lame cows, based on repeated mobility scores throughout this project, ranged from 18.8 to 33.3% across the 4 herds; the mean point prevalence of lameness from all timepoints ranged from 6.1 to 12.0% across the 4 herds. Recent cross-sectional studies in the UK reported that herd lameness prevalence ranges from 6 to 65%; this suggests the 4 herds in our study have a lower prevalence of lameness than many dairy herds in the UK (Griffiths et al., 2018; Randall et al., 2019). We observed the peak prevalence of sole lesions to be in early lactation (T3-Early) when 21.4% of cows had severe sole hemorrhage and 6.1% of cows had sole ulcers. The prevalence of sole lesions in the peer-reviewed literature has historically only been reported in large numbers for lame animals or from foot-trimming records. Therefore, previous reports may not have a reliable numerator (due to underreporting of mild lesions) or a reliable denominator (due to over-representation of lame cows). In studies using foot-trimming records, the prevalence of sole hemorrhage has been reported to range from 5 to 59% (Capion et al., 2008; Malchiodi et al., 2017), and for sole ulcers from 5 to 17% (van der Waaij et al., 2005; König et al., 2008).

## CONCLUSIONS

The results from this prospective cohort study indicate that recovery from sole lesions is a heritable trait in Holstein cows. This result requires replication in further studies; however, the potential may exist to selectively breed cows that can recover more effectively from sole lesions. Our results suggest that recovery from sole lesions is only weakly genetically correlated with overall susceptibility to sole lesions, although this finding also requires corroboration. If sole lesion susceptibility and recovery are only weakly genetically correlated, selecting for resistance to sole lesions may have a limited impact on the ability of affected cows to recover, and a recovery trait would need to be evaluated specifically. Additionally, the apparent weakness of the genetic correlation between sole lesion susceptibility and recovery has interesting biological implications because the genetic background to each trait could be inferred to be largely independent.
